# Improvement of GNSS Carrier Phase Accuracy Using MEMS Accelerometer-Aided Phase-Locked Loops for Earthquake Monitoring

**DOI:** 10.3390/mi8060191

**Published:** 2017-06-19

**Authors:** Tisheng Zhang, Hengrong Liu, Qijin Chen, Hongping Zhang, Xiaoji Niu

**Affiliations:** GNSS Research Center, Wuhan University, 129 Luoyu Road, Wuhan 430079, China; zts@whu.edu.cn (T.Z.); liuhengrong163@163.com (H.L.); hpzhang@whu.edu.cn (H.Z.); xjniu@whu.edu.cn (X.N.)

**Keywords:** PLLs, MEMS accelerometer, carrier phase, GNSS, earthquake monitoring

## Abstract

When strong earthquake occurs, global navigation satellite systems (GNSS) measurement errors increase significantly. Combined strategies of GNSS/accelerometer data can estimate better precision in displacement, but are of no help to carrier phase measurement. In this paper, strong-motion accelerometer-aided phase-locked loops (PLLs) are proposed to improve carrier phase accuracy during strong earthquakes. To design PLLs for earthquake monitoring, the amplitude-frequency characteristics of the strong earthquake signals are studied. Then, the measurement errors of PLLs before and after micro electro mechanical systems (MEMS) accelerometer aiding are analyzed based on error models. Furthermore, tests based on a hardware simulator and a shake table are carried out. Results show that, with MEMS accelerometer aiding, the carrier phase accuracy of the PLL decreases little under strong earthquakes, which is consistent with the models analysis.

## 1. Introduction 

Global navigation satellite systems (GNSS), as a primary technique of modern geodetic observation, can measure accurate crustal deformation. For a large seismic event, high-rate GNSS can provide rapid estimates of broadband displacements, including static offsets and dynamic motions of arbitrarily large magnitude [1,2,3,4]. However, the errors of GNSS measurements during dynamics are systematically larger than those during static periods [5,6,7]. GNSS-derived dynamic motions by themselves are not accurate enough to identify millimeter-level or even smaller amplitude P-waves [8,9]. 

The strong-motion accelerometer has a superior dynamic characteristic, which is highly complementary to GNSS. Some combined processing strategies have already been presented [9,10,11,12,13,14]. Bock et al. applied a loosely coupled Kalman filter to combine high rate GNSS displacements and micro electro mechanical systems (MEMS) accelerometer data in near real time to estimate better precision in displacement. Geng et al. estimated the displacement and velocity from GNSS phase observations and accelerations using a tightly coupled Kalman filter approach. These data combined strategies have no help on the accuracy improvement of GNSS carrier phase, which would deteriorate the displacement measurement precision.

GNSS carrier phase is generated by the receiver’s carrier tracking loops [15]. The tracking bandwidth and integration time play an important role on accuracy and dynamic stress tolerance [16,17]. To reduce the noise and improve accuracy, the tracking bandwidth should be narrow and the integration time should be long. However, to be better suited for dynamic stress, wider tracking bandwidths and shorter integration times are ideal [18,19,20]. This causes a design contradiction of carrier tracking loops for earthquake monitoring. The majority of research on GNSS receivers for earthquake monitoring has been on the advantages of their use. There has been little research related to improving receiver measurements performance. Adam et al. designed an adaptive tracking loop to overcome the design contradiction of high accuracy and dynamic stress tolerance requirements [21]. The performance of phase-locked loops (PLLs) with different integration time and bandwidth are compared under earthquake dynamics. It showed that, to obtain optimal performance, the integration time should be set as 10 ms, and the bandwidth need be changed with the dynamics. The adaptive tracking loop is capable of providing low noise before the start of an earthquake with narrow bandwidth. However, when the earthquake occurs, the tracking bandwidth would be wide enough to tolerance dynamics, which would increase the noise of carrier phase. Therefore, we focus on solving carrier phase accuracy impacted by the bandwidth.

In navigation field, GNSS and the inertial navigation system (INS) deep couple has been studied to improve tracking loops performance under dynamic environments [22,23]. To improve the GNSS carrier phase accuracy on earthquake monitoring, the GNSS/MEMS accelerometer deep couple must be studied. The objective of this research is to improve GNSS carrier phase accuracy using MEMS accelerometer-aided phase-locked loops (PLLs) for earthquake monitoring. The amplitude-frequency characteristics of the strong earthquake signals are studied first. Then, the measurement errors of PLLs before and after MEMS accelerometer aiding are analyzed. Furthermore, the tests based on a hardware simulator and a shake table are carried out. Results and their analysis are then presented. Finally, conclusions are shown.

## 2. Characteristics of Strong Earthquake Signal

**Figure 1:**
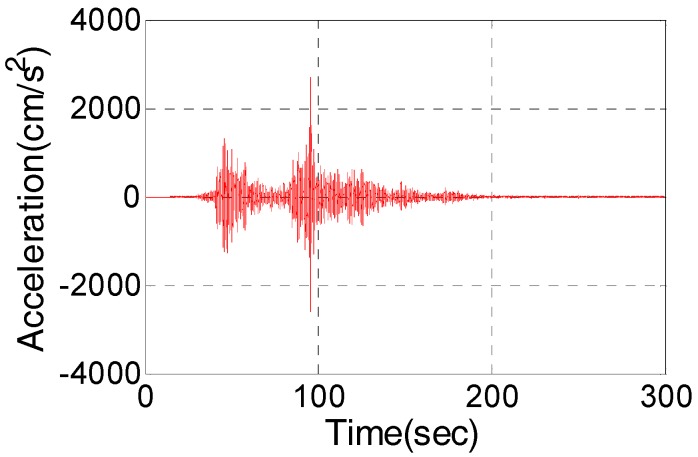
Acceleration sequence from 2011 Honshu earthquake.

To design the PLLs of a GNSS receiver, used for strong earthquake monitoring, we shall find out the motion characteristics of strong earthquake signal. Taking the 2011 M9.0 Honshu earthquake in Japan as an example, its acceleration sequences in three axes were recorded by the MYG004 station during the earthquake period. Figure 1 shows the acceleration sequence in the north-south (N-S) direction. It can be seen that the earthquake lasted more than 250 s, and the maximum acceleration in the N-S direction exceeds 2 g, which is a typical strong earthquake wave. The maximum acceleration will provide the benchmark for performance testing of PLLs.

**Figure 2:**
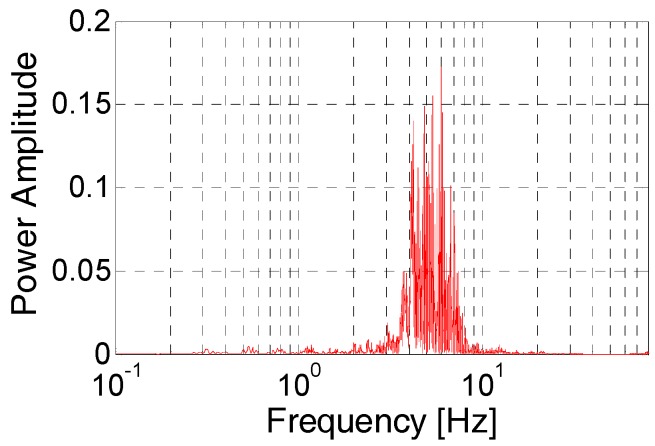
Power spectrum of acceleration data from 2011 Honshu earthquake.

The power spectral density of acceleration data is useful for understanding the range of frequencies in strong earthquake signals [24]. The acceleration sequence is processed using the earthquake wave processing software SeismoSignal [25]. The power spectral density is shown in Figure 2. It reflects the power distribution on each frequency of the earthquake signals. The majority of the acceleration in the N-S direction is in the range of 0 to 10 Hz.

Since the earthquake has a large amplitude and high frequency acceleration, the GNSS receiver’s design must be targeted to improve the carrier phase accuracy. Based on the characteristics of a strong earthquake signal, typical sine wave motion was set on the simulator, and a shake table was used to simulate strong earthquakes in our research.

## 3. Measurement Errors of PLLs

Based on the tracking error model of stand-alone PLLs, the impacts of the signal strength and the PLL bandwidth on the carrier phase accuracy are analyzed under strong earthquake dynamics. Then, the tracking error model of MEMS accelerometer-aided PLLs is discussed, and the improvement to carrier phase measurement under strong earthquake dynamics is analyzed.

### 3.1. Stand-Alone PLLs

**Figure 3:**
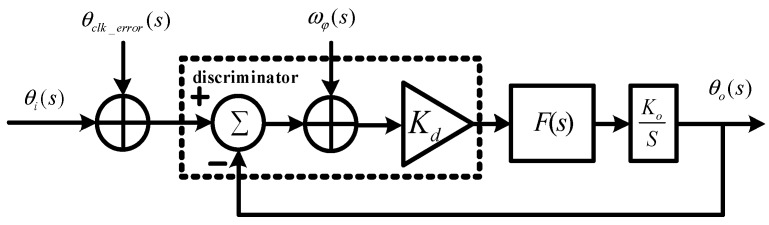
Error transfer model of stand-alone phase-locked loop (PLL).

The signal transfer relationship of the stand-alone loop in a Laplace domain is shown in Figure 3. The tracking loop includes a discriminator, a low-pass filter F(s), while a numerically controlled oscillator (NCO) $K_{o}/S$. $\theta_{i}\left(s\right)$ is the input signal, and $\theta_{0}\left(s)\right)$ is the output signal. $\omega_{\varphi }\left(s\right)$ is the thermal noise in the tracking loops. $\theta_{clk\_error}\left(s\right)$ is the local oscillator error, added to $\theta_{i}\left(s\right)$ by down conversion. In addition, the dynamic stress also leads to tracking error. Therefore, the error sources of the carrier phase include thermal noise $\sigma_{tPLL}$, vibration-induced oscillator jitter $\sigma_{rv}$, Allan variance-induced oscillator jitter $\sigma_{rA}$, and dynamic stress $\theta_{e}$. Since all error sources are independent, the mean squared error of total errors can be written as follows:

(1) $$\sigma_{PLL}=\sqrt{\sigma_{tPLL}^{2}+\sigma_{rv}^{2}+\sigma_{rA}^{2}}+\theta_{e}/3$$

Taking the second-order PLL as an example, the models of the error sources can be expanded as follows [26]:

(2) $$\sigma_{tPLL}=\frac{180}{\pi }\sqrt{\frac{B_{n}}{C/N_{0}}\cdot \left(1+\frac{1}{2T_{coh}\cdot C/N_{0}}\right)}\left({}^{\circ}\right)$$

(3) $$\sigma_{rv}=\frac{180}{\pi }\sqrt{\frac{\pi^{2}{f_{0}}^{2}{K_{g}}^{2}G_{g}}{2.67B_{n}}}\left({}^{\circ}\right)$$

(4) $$\sigma_{rA}=\frac{180}{\pi }\sqrt{2\pi^{2}{f_{0}}^{2}\cdot \left(\frac{\pi^{2}h_{-2}}{\sqrt{2}\left(1.89B_{n}\right)}+\frac{\pi h_{-1}}{4{\left(1.89B_{n}\right)}^{2}}+\frac{h_{0}}{4\sqrt{2}\left(1.89B_{n}\right)}\right)}\left({}^{\circ}\right)$$

(5) $$\theta_{e}=\frac{\Delta ℜ}{{\left(1.89B_{n}\right)}^{2}}\left({}^{\circ}\right)$$

where $B_{n}$ is the noise bandwidth, $C/N_{0}$ is the carrier-to-noise ratio (CN0), $T_{coh}$ is the coherent integration time, $f_{0}$ is the carrier frequency, $K_{g}$ is oscillator sensitivity, $G_{g}$ is the single-sided vibration spectral density, $h_{i}$ is a constant, and Δℜ is the frequency ramp-up amplitude. The Costas PLL is insensitive to phase inversion of 180°. When $\sigma_{PLL}$ is larger than the threshold 15°, the PLL loses lock.

**Figure 4:**
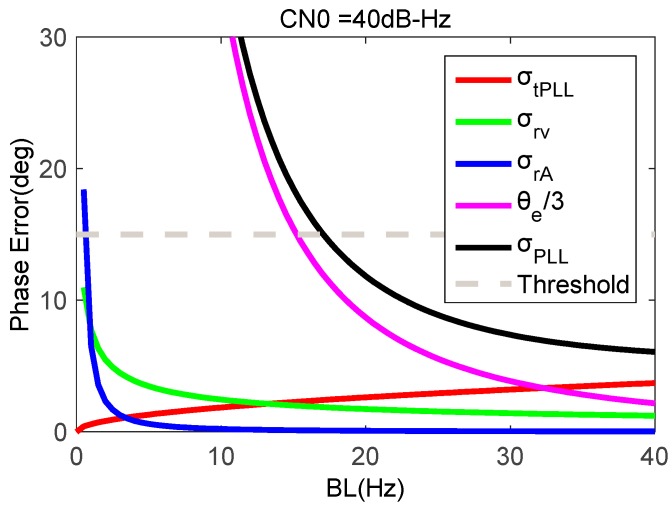
Phase errors of second-order PLL as a function of bandwidth.

Based on above error model, the impacts of GNSS signal strength and the bandwidth of tracking loops on tracking error can be analyzed under strong earthquake motion. An oven-controlled crystal oscillator (OCXO) is used as our receiver clock, with parameters that $h_{-2}=2.51\times 10^{-22},h_{-1}=2.51\times 10^{-23},h_{0}=2.51\times 10^{-26}$. When the signal CN0 is 40 dB-Hz, the movement acceleration is 2 g, and the coherent integration time of PLL is set as 1 ms, Figure 4 shows tracking errors as a function of the PLL bandwidth. The tracking error caused by thermal noise increases with the increase of the bandwidth. Since the signal with a CN0 of 40 dB-Hz is a strong signal, the tracking error increases slowly with bandwidth increasing. Tracking errors caused by the oscillator and dynamic stress decrease with the increase of the bandwidth. Because the tracking error caused by dynamic stress changes significantly larger than others, it is necessary to increase the bandwidth to reduce the total tracking error appropriately. To ensure the total loop tracking error less than the threshold, the bandwidth must be wider than 17 Hz.

**Figure 5:**
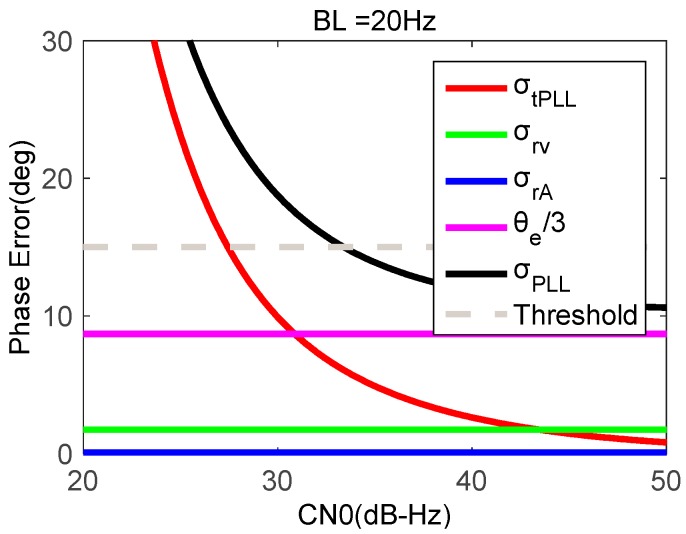
Phase errors of second-order PLL as a function of signal strength.

When the movement acceleration maintains 2 g, the PLL bandwidth is 20 Hz, and the coherent integration time is 1 ms, Figure 5 shows tracking errors as a function of signal CN0. As is seen in Figure 5, the errors caused by dynamic stress and oscillator are in dependent of GNSS signal strength. With the signal CN0 reducing, the tracking error caused by thermal noise increases greatly. When the CN0 is less than 34 dB-Hz, the total phase error exceeds the tracking threshold. Hence, when the GNSS signal is weak under dynamics, the bandwidth should be compromised. Therefore, the carrier phase accuracy from the stand-alone PLLs deteriorates under strong earthquake motion.

### 3.2. MEMS Accelerometer-Aided PLLs

**Figure 6:**
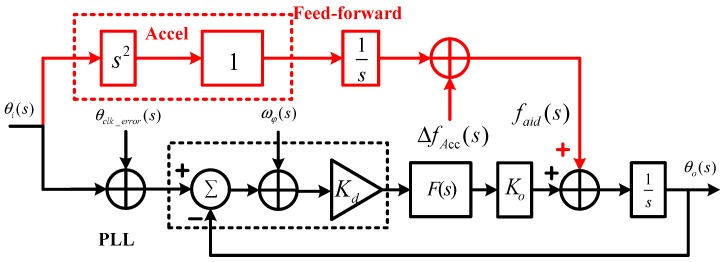
Error transfer model of microelectromechanical systems (MEMS) accelerometer-aided PLLs.

When a strong earthquake occurs, the GNSS receiver and the MEMS accelerometer at the same location bear the same dynamic stress. Hence, the dynamic information measured by the MEMS accelerometer can aid GNSS tracking loops. The signal transfer relationship of the MEMS accelerometer-aided PLLs in a Laplace domain is shown in Figure 6. Compared with Figure 3, the MEMS accelerometer information assists PLLs based on the concept of feed-forward [27]. The velocity is obtained by the integration of the accelerometer measurement, and maps to the line of sight (LOS) between the satellite and receiver to obtain the Doppler estimation information $f_{aid}\left(s\right)$ [28]. With the aid of the accelerometer feed-forward information, the tracking loop only needs to undertake the error of the aiding information $\Delta f_{A\mathrm{cc}}(s)$, instead of dynamic stress.

For the GNSS/MEMS accelerometer deeply coupled system, the error sources of the aiding information include the initial velocity error $\delta V_{N}\left(0\right)$ after correction of GNSS/accelerometer integration update, the accelerometer bias, the accelerometer scale factor, etc. Our previous research showed that $\delta V_{N}\left(0\right)$ was the main factor of the aiding information impacting the PLL phase error [29]. Based on the transfer relationship between the aiding information error and the loop tracking error, the loop tracking error caused by the aiding information in time domain can be deduced as follows:

(6) $$\sigma_{faid}=\frac{180}{\pi }\sqrt{\frac{2\pi \cdot \delta V_{N}\left(0\right)}{\lambda \cdot e\cdot \omega_{n}}}\left({}^{\circ}\right)$$

where λ is the carrier wavelength, and $\omega_{n}$ is the natural radian frequency. $\sigma_{faid}$ is a random constant value in each update interval and follows the Gaussian distribution. Since $\sigma_{faid}$ is uncorrelated with that due to thermal noise and oscillator noise, the error caused by aiding information is the geometric sum relationship to that caused by thermal noise and oscillator. Therefore, compared with Equation (1), the mean squared error of total errors of MEMS accelerometer-aided PLL can be written as follows:

(7) $$\sigma_{PLLaid}=\sqrt{\sigma_{tPLL}^{2}+\sigma_{rv}^{2}+\sigma_{rA}^{2}+\sigma_{faid}^{2}}$$

**Figure 7:**
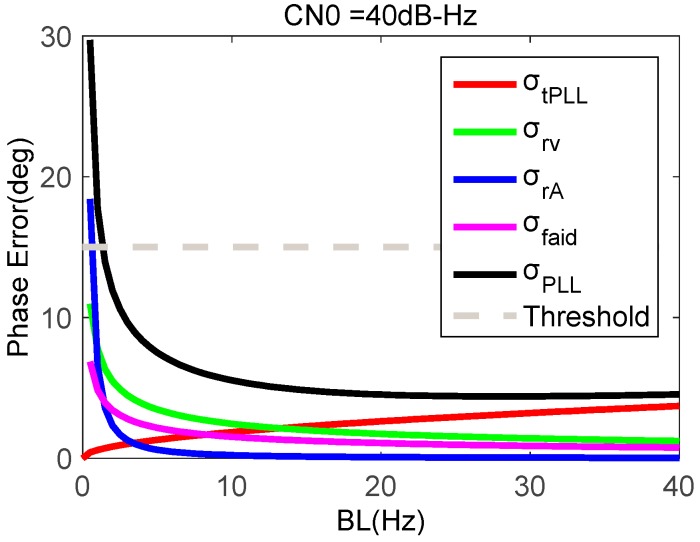
Phase errors of MEMS accelerometer-aided PLL with a carrier-to-noise ratio (CN0) of 40 dB-Hz.

Taking a typical MEMS sensor STIM 300 as an example [30], we obtain the statistic of $\delta V_{N}\left(0\right)$ based on field tests, which is about 0.01 m/s. Based on above error model, the phase error of MEMS accelerometer-aided PLLs can be analyzed. When the movement acceleration is 2 g, the CN0 is 40 dB-Hz, and the coherent integration time of the PLL is 1 ms, Figure 7 shows phase errors of MEMS accelerometer-aided PLL as a function of PLL bandwidth. Compared with Figure 4, the phase error caused by the aiding information is much less than that caused by the dynamic stress, which also increases with the bandwidth narrowing. The impact of the thermal noise and crystal oscillator keep the same. The accelerometer-aided PLL with a bandwidth of more than 2 Hz can keep the total phase error less than the threshold, which is much narrower than the stand-alone loop bandwidth. And with the accelerometer aiding, the total phase error significantly reduces.

**Figure 8:**
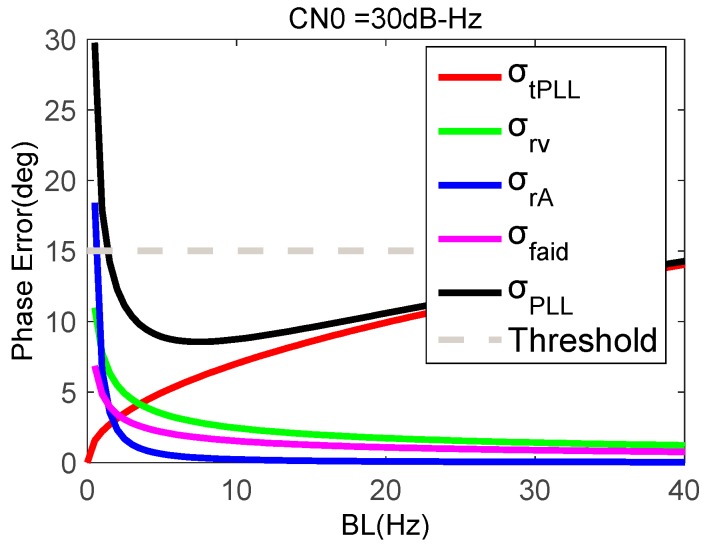
Phase errors of MEMS accelerometer-aided PLL with a CN0 of 30 dB-Hz.

When the strong earthquake occurs, the GNSS signal may become weak. To obtain an accurate and stable carrier phase under a strong earthquake, it is necessary to study the relationship between the loop bandwidth and the tracking error under a weak signal. Figure 8 shows phase errors as a function of PLL bandwidth when the CN0 is down to 30 dB-Hz. Compared with Figure 7, the phase error caused by thermal noise clearly increases. When the loop bandwidth is 5 Hz, the total phase error is the smallest.

We summarize the measurement error of PLLs as follows. Under strong earthquake dynamics, the bandwidth of the stand-alone PLLs cannot be less than 17Hz, and the phase measurement error increase with GNSS signal fades. MEMS accelerometer-aided PLLs with narrow bandwidth can track GNSS signal with little phase error under strong earthquake dynamics. Above error models are helpful to the analysis of phase error and the selection of optimal bandwidth.

## 4. Carrier Phase Accuracy Tests

Based on the characteristics of strong earthquake signal, a motion case with sinusoidal acceleration with a frequency of 2 Hz, and a magnitude of 2 g, was set on the simulator, and a shake table was used to simulate a strong earthquake. The carrier phase measurement accuracy of the stand-alone second-order PLL, stand-alone third-order PLL, and MEMS accelerometer-aided second-order PLL were compared based on the simulator and shake table, respectively.

### 4.1. Tests and Analysis Based on Signal Simulator

A GNSS/inertial hardware signal simulator can generate typical scenarios with strict repeatability, flexible parameter set, less external disturbance and, most importantly, with perfect true values for error analysis. It is an effect method to test the performance of PLLs under strong earthquake dynamics. According to the amplitude-frequency characteristics of the strong earthquake signal, a simulated strong earthquake dynamic trajectory was set on the signal simulator. And the GNSS/MEMS accelerometer raw date was processed in our deeply coupled software system to test PLL performance.

**Figure 9:**
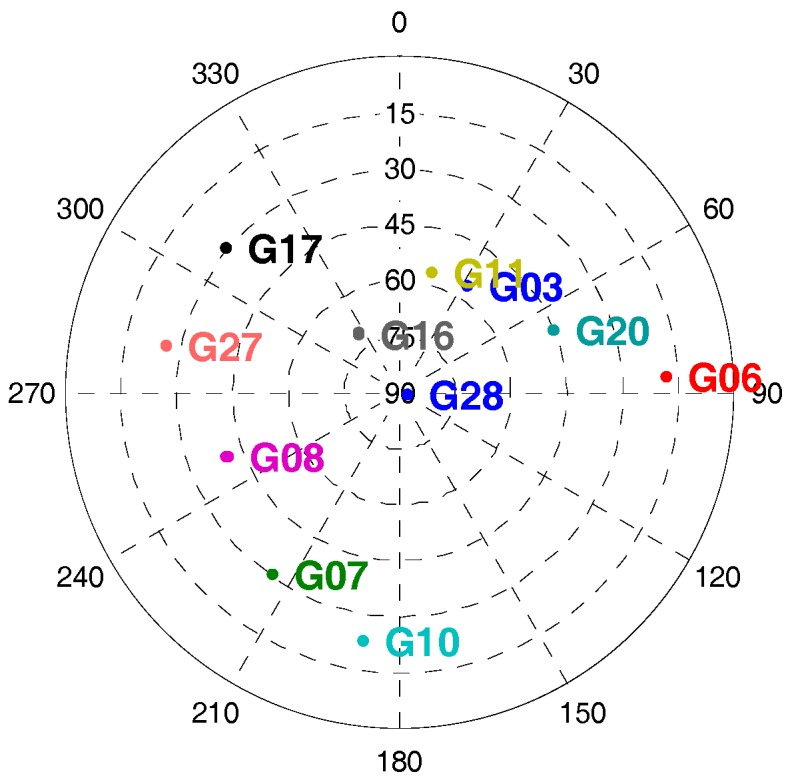
Satellite sky distribution in signal simulator tests.

**Figure 10:**
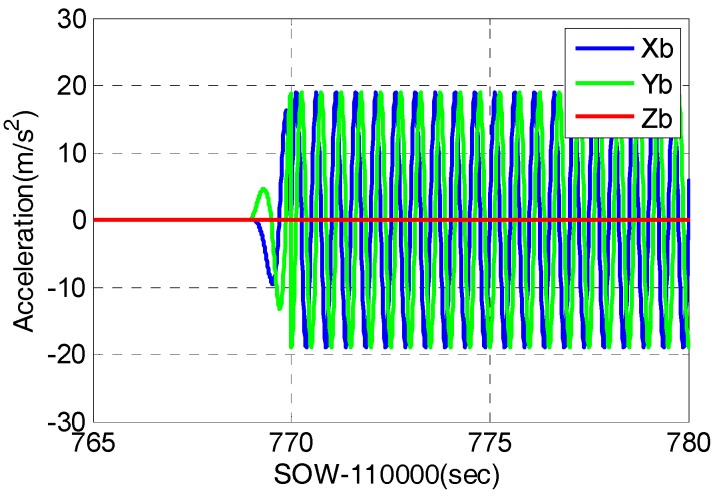
Recorded accelerometer raw data in signal simulator tests.

The typical strong earthquake signal characteristics showed that the maximum acceleration was more than 2 g and its power spectral density was in the range of 0 to 10 Hz. Therefore, a motion case with sinusoidal acceleration was set on the simulator with a frequency of 2 Hz, and a magnitude of 2 g. In this motion case, the receiver was initially static for some time, and then did circular motion with only translational movement, and no angular movement in the horizontal plane for 100 s. The accelerometer parameters set was based on STIM300. The GNSS signal strength was set as 45 dB-Hz, and the GNSS satellite distribution was shown in Figure 9. Since the elevation of satellite G10 was low, the dynamic between receiver and G10 was large, which was about 1.7 g. Figure 10 showed the recorded accelerometer raw data, which was consistent with simulator set.

Taking satellite G10 as an instance, the stand-alone PLL and MEMS accelerometer-aided PLL were tested and analyzed on our deeply coupled software system. Since the output of PLL discriminator can reflect the carrier phase accuracy of the PLL, it was used to evaluate the PLL performance.

**Figure 11:**
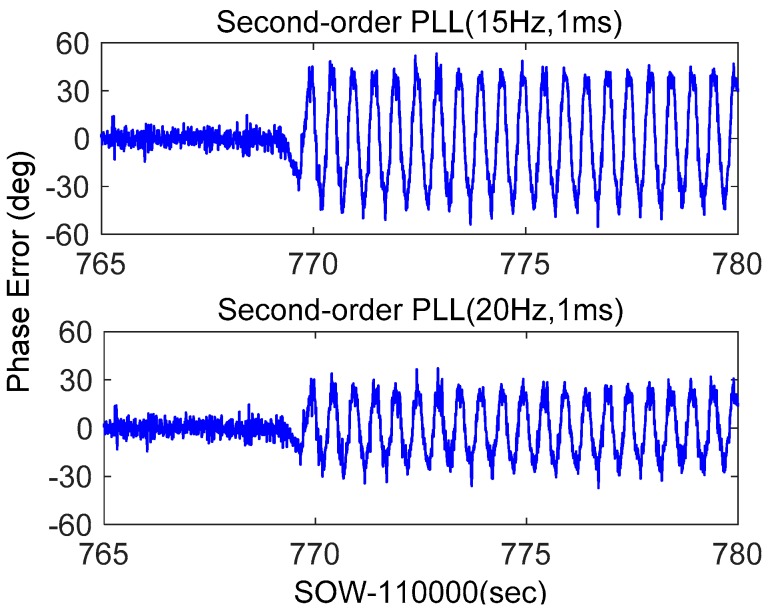
Phase error of stand-alone second-order PLL.

Stand-alone second-order PLLs with bandwidths of 15 Hz and 20 Hz were tested. Their discriminator outputs were shown in Figure 11. When the receiver was static, the discriminator outputs were less than 10°, mainly caused by thermal noise and oscillator. When the movement happened, the maximum discriminator output of PLL with a bandwidth of 15 Hz was about 40°. When the loop bandwidth was increased to 20 Hz, the maximum discriminator output reduced to 24°. These results were consistent with the theoretical values calculated by Equation (5). Testing results showed that the phase errors of stand-alone PLLs had a significant increase under dynamics, and the phase errors caused by the dynamic stress could be reduced by widening the bandwidth. However, considering the impact of a weak satellite signal, discussed based on Figure 5, the stand-alone PLL bandwidth cannot be increased blindly.

**Figure 12:**
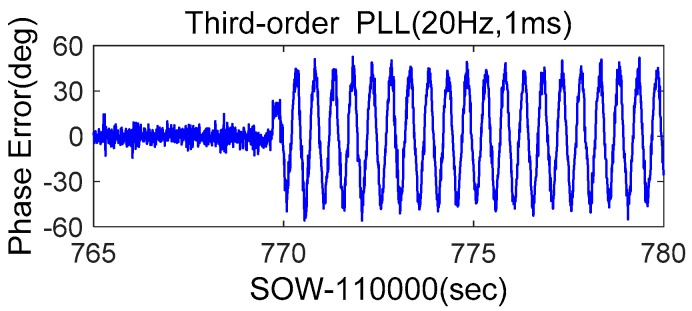
Phase error of stand-alone third-order PLL.

Since third-order PLLs can track higher order dynamics, most commercial receivers use third-order PLLs. Therefore, a third-order PLL with a bandwidth of 20 Hz was tested. Its discriminator output was shown in Figure 12. When the movement occurred, the maximum discriminator output exceeded 40°, clearly higher than that of the second-order PLL with the same bandwidth. This is because the acceleration frequency is bigger than 1 Hz, which caused the amplitude of jerk to be larger than that of acceleration. Therefore, for a strong earthquake application, third-order PLLs have no advantage.

**Figure 13:**
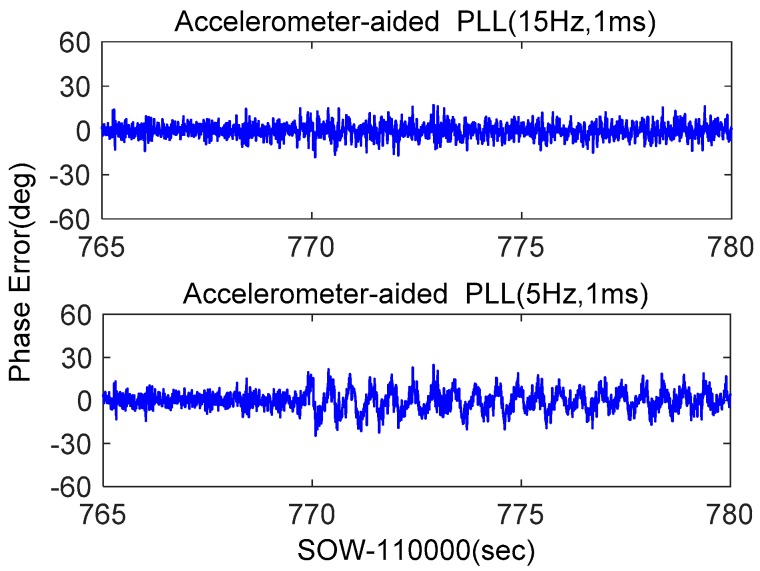
Phase error of accelerometer-aided second-order PLL.

MEMS accelerometer-aided second-order PLLs with bandwidths of 15 Hz and 5 Hz were tested. Their discriminator outputs were shown in Figure 13. When the loop bandwidth is 15 Hz, the phase error almost had no increase under dynamics. Compared with Figure 11 and Figure 12, the phase error caused by dynamics was almost eliminated with accelerometer aiding. When the loop bandwidth was compressed to 5 Hz, the static error was not significantly reduced. This is because the simulation signal was strong, which only caused small phase error, consistent with the analysis in Figure 5. When the dynamics occur, the phase error of PLL with a bandwidth of 5 Hz is larger than that of 15 Hz. This is caused by the time delay of accelerometer data recording, whose impact increases with bandwidth narrowing. This phenomenon was analyzed in detail in our previous research [31]. The simulation results showed that the accelerometer-aided PLL could weaken the impact of dynamics on the measurement accuracy of carrier phase, which was helpful to improve GNSS accuracy under strong earthquakes.

### 4.2. Tests and Analysis Based on a Shake Table

**Figure 14:**
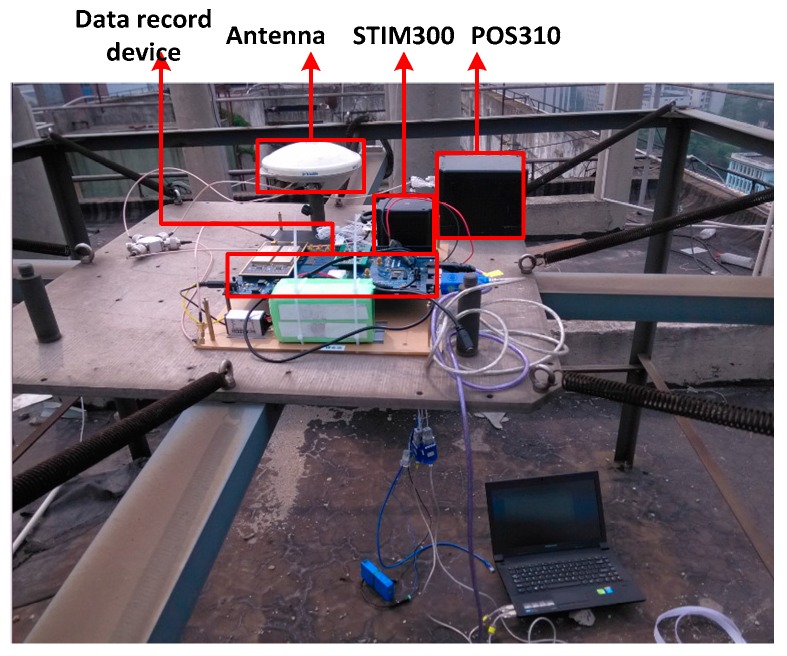
Tests set based on a shake table.

The shake table is another tool to simulate strong earthquakes. The carrier phase measurement accuracy was further verified on the shake table, shown in Figure 14. The GNSS antenna, MEMS accelerometer STIM300, raw data recording device and reference system POS310 were fixed on the shake table. POS310, a high-precision GNSS/INS coupled system, was used to provide dynamic reference value. STIM300 raw data was used to aid GNSS signal tracking. The lever arms between the GNSS antenna and inertial devices were measured and compensated in a data fusion algorithm.

**Figure 15:**
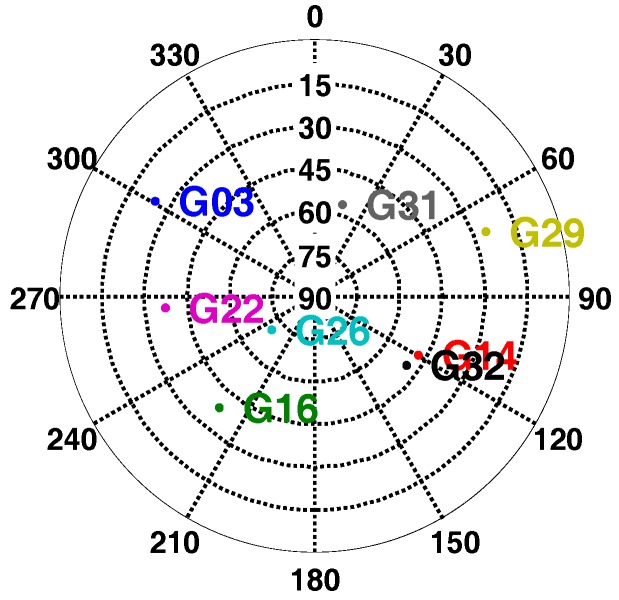
Satellite sky distribution in shake table tests.

**Figure 16:**
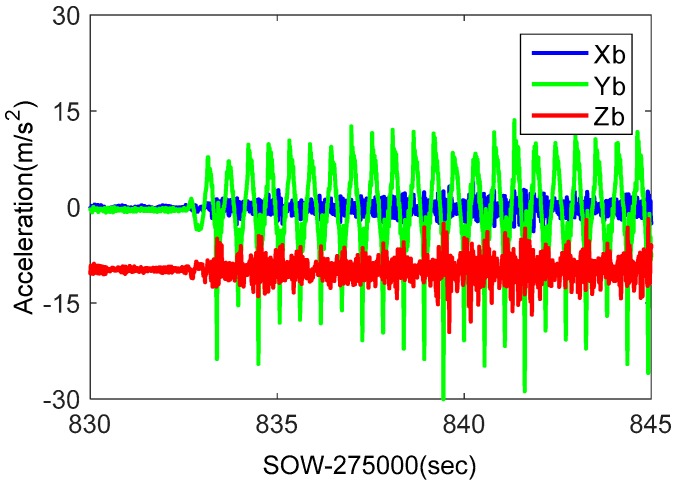
Recorded accelerometer raw data in shake table tests.

The shake table was static for some time, and then shook for 30 s in the horizontal direction. Figure 15 showed the GPS satellites distribution during the shake table tests. G29, a low elevation angle satellite, was to analyze the impact of shake at horizontal direction. Additionally, G26, a high elevation angle satellite, was to analyze the impact of shake at vertical direction. The acceleration information measured by POS310 was shown in Figure 16. The main frequency of acceleration was about 2 Hz, and the maximum acceleration value was larger than1g.

**Figure 17:**
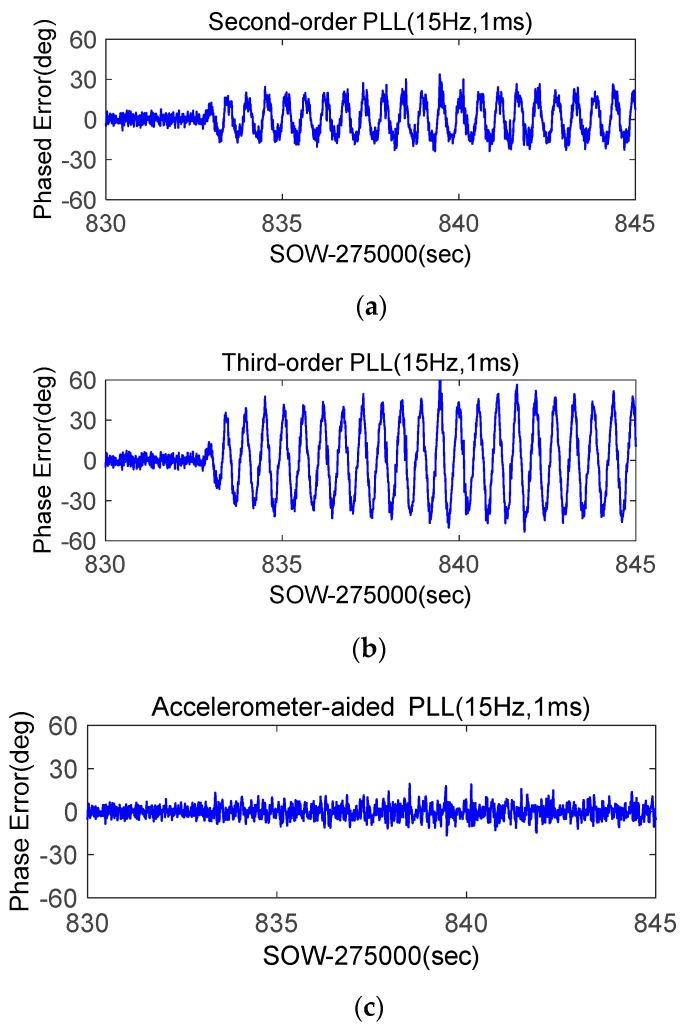
Phase errors of low elevation angle satellite G29 based on shake table tests; (**a**) Phase error of second-order PLL; (**b**) Phase error of third-order PLL; (**c**) Phase error of accelerometer-aided second-order PLL.

**Figure 18:**
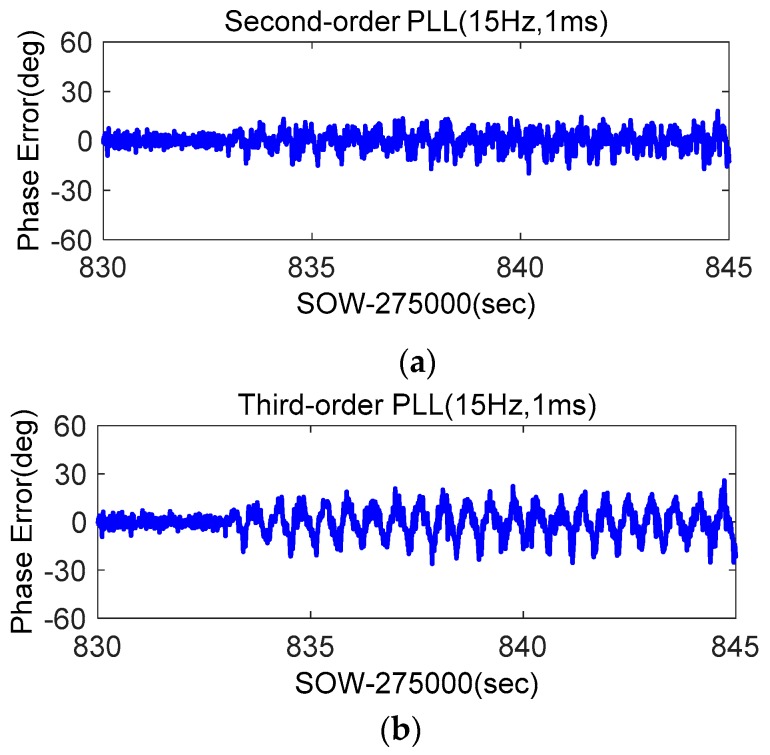

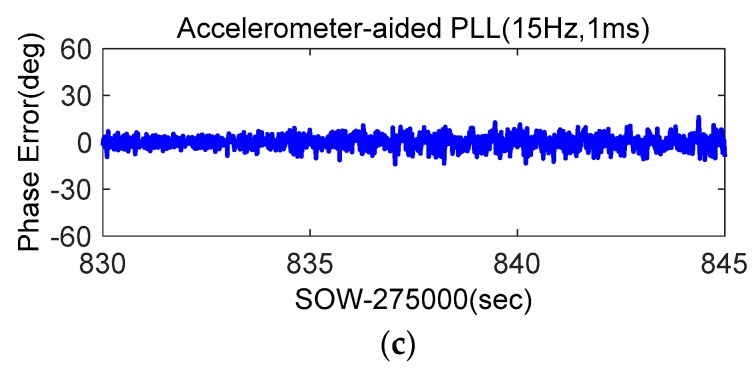
Phase errors of high elevation angle satellite G26 based on shake table tests; (**a**) Phase error of second-order PLL; (**b**) Phase error of third-order PLL; (**c**) Phase error of accelerometer-aided second-order PLL.

Phase errors of stand-alone second-order PLL, stand-alone third-order PLL, and STIM300-aided second-order PLL were compared. To reduce the impact of the accelerometer data recording delay, their bandwidths were all set as 15 Hz, and integration times were set as 1 ms. The results of G29 were shown in Figure 17. When the shake occurs, the phase error of the stand-alone second-order PLL was more than 20°, that of the third-order PLL was more than 40°, and the STIM300-aided second-order PLL was only slightly larger than that of the static. The testing results were consistent with the model analysis and simulator testing results. Figure 18 showed the results of G26. Since the dynamics at vertical direction were smaller than that at horizontal direction, the phase error of G26 was smaller. With the STIM300 aiding, its phase error caused by dynamics also had a big reduction. Shake table tests verify once more that the MEMS accelerometer aiding can effectively improve the GNSS carrier phase measurement accuracy under strong earthquake.

## 5. Conclusions

To improve GNSS carrier phase measurement accuracy under strong earthquakes, MEMS accelerometer-aided PLL is proposed. The carrier phase errors of stand-alone PLL and MEMS accelerometer-aided PLL are analyzed based on their error models. Under strong earthquake dynamics, the stand-alone PLLs with a bandwidth narrower than 17Hz lose lock, but MEMS accelerometer-aided PLLs with a bandwidth of 5 Hz can track a 30 dB-Hz signal with small carrier phase error. Simulated strong earthquake tests are carried out on the simulator and shake table. Test results show that, while the carrier phase errors of stand-alone PLLs are larger than 20°, MEMS accelerometer-aided PLL errors are smaller than 5°. The proposed MEMS accelerometer-aided PLL can effectively improve the GNSS carrier phase measurement accuracy under strong earthquakes. The improvement of the displacement precision could be analyzed in future.
